# Music

**Published:** 1882-10

**Authors:** Ausburn Towner


### MUSIC.

AUSBURN TOWNER.

Did you, who read these lines, and cannot remember the time when similar ones were not intelligible to you, did you ever think how strange these odd little shapes, arbitrarily named and called letters, grouped into words, must appear to those persons who can not read? To you, the pages of a book talk silently as though they had tongues; to those, they are mere scraps of paper, spattered over with regular and shapely, but meaningless blots of ink. If you would get some idea of how it would be with you, just take a page of Egyptian hieroglyphs or of Hebrew text, and see what a wilderness of scratches it seems to be, redeemed by the thought, that, although it makes not the slightest impression upon your intelligence, to those who can understand it, it has a meaning. In much the same way, must musical notation appear to those who are not educated to read it. The five horizontal parallel lines with their upright posts at intervals like a well-built fence; the round, head-like looking objects staring white or black at you from between the lines or upon them; some of them without legs; some with one leg and others with a multitude of arms all thrown one way; others, seeming, with a very little effort of the imagination to be little negro children trying to climb over a fence; others having steps built up on purpose for them to go higher than the upper line, into the attic and on the house-top perhaps, or lower than the lowest line, down into the cellar or farther below perhaps; all of these are more or less familiar to the public eye. Every one knows what they are intended for. But it is only to the fortunate and favored few that they address themselves intelligently, and to the still less number do they carry a sense of melody or harmony or what we generally call music, except with the aid of an instrument or the voice. A deaf and dumb man may be able to read a poem that is full of beauty and get it all in detail, scope and total meaning, enjoying it to the utmost and having his heart filled with the thoughts it contains. How high the culture, how rich the soul of that one, who, in like manner, is so attuned that, by the aid of his eye alone, he can get from a printed or written score, the sentiment and meaning of its author, or its character. There are a few, only a few persons, you can count them on your fingers, in this city, who are, in a measure gifted in this manner, or who, by arduous and constant study, have obtained such a power. The great Beethoven, stricken with deafness, after he had reached the age of forty years, possessed it in a remarkable degree. What a blank would have been his life without it! He could feel and understand a score without being able to hear it performed. To the majority of mankind, these few persons, in situation, not in numbers, are, what the majority of the civilized world, are to those who can not read. What a world of satisfaction, joy and gladness are those shut out from! Years ago, when only the very few knew how to read, they were doubtless looked upon as many now regard those who, at first sight, can interpret to themselves or to others, if necessary, a musical work. Yet that which we generally understand by the term music goes away back in the past. Its origin must have been cotemporaneous with language, if not ante-dating it, as the crooning and murmured monotone of an infant comes days and weeks before it can utter a syllable. It would have seemed that the fixing of a melody or tune by means of arbitrary characters, or methods of writing music would have long preceded the invention of letters to express the spoken word. There is something in the rising and falling of the voice in its measured cadences, inflections and emphases that suggest at once a method to express them by lines, heavy or light, rising suddenly or abruptly by a perpendicular, or gradually by an inclined line or curve and then dropping with a similar expression to a normal pitch. All of the means, so far as we know, to express music by writing are entirely arbitrary, the signs, in themselves suggesting nothing, and that at present in use, not the least so of any. Thirty years or so ago, some musician in this country endeavored to introduce a system, in which the figures up to seven, in various positions, stood for the several notes. But his attempt was a failure, and he and it are forgotten except by a few. The foundation of the method in use is indeed very simple, but the numberless combinations possible render it sometimes very intricate and difficult.

The ancient Greeks wrote their music, such as they had, by means of the letters of their alphabet placed in various positions, mutilated and compounded in such a multitude of ways that there were more than a thousand signs and characters employed. The manner in which it was read has been lost, so that we are unable to tell what their music was like. It is no wonder that in the education of their youth three years were spent in the study of the divine art, for at least that length of time must have been necessary to teach the pupils the meaning of the numerous characters, so that they could be used with any sort of rapidity or accuracy. Nevertheless, there are records showing that there were many then skilled in music; that for this they were highly regarded and were paid enormous prices for their public performances. It is related that a flute player of Athens, named Amœbæus received one talent, what would now be nearly $1,000, for a single appearance at the theater, and that other players on a like instrument lived in magnificent style from the proceeds of their entertainments. The name of one fair lady has come down to us, through all these years, simply and only from her proficiency in playing the flute. This was Lamia, the daughter of Cleanora. Her flute playing, taken with her wit and beauty, caused her to be esteemed something of a prodigy. These and other instances like them make seem less singular similar ones in these modern days when Jenny Lind, Patti, Nillson or Rubenstein obtain large sums and easily win great fortunes by the exercise of their musical genius.

So far as we are concerned we might, with much greater show of reason than did the ancients, attribute to music a divine origin, for we are certainly indebted to our religion for what it is to us now. In compiling a musical book for the use of churches it has been related that Henry Ward Beecher, in selecting certain tunes, observed, in substance, that it was not right for the devil to have all the best music, thus intimating that the person or spirit named was the instigator, or at the bottom of the science of the concord of sweet sounds. On the contrary, he stole from the church all that he or any of his devotees know of music, as he has stolen the drama and many another noble idea, claiming it for his own and putting it to his uses.

Music, such as we have now, had its origin in the early Christian church. It was not borrowed from the Greeks or Romans, but came from the Jews. So long ago as the year 386, less than four hundred years after our Saviour was on earth, St. Ambrose, the Bishop of Milan, made a great reformation in the music of the church. There must, therefore, have been music before that time, else it could not have been reformed. What kind of music we can only conjecture, as there are no remains of it left, but it was the foundation of the Gregorian style which was introduced by the Pope of that name not quite two hundred years after St. Ambrose, and which is yet sung in the Catholic churches of the world. What strength, what power, what beauty must there be in a combination of sounds that have been sung in the worship of God more than thirteen hundred years! An inspiration must have been the origin of such music; it must have been given to the one who produced it by a higher power and by no volition of his own. It is easy from such instances to understand and believe in the inspiration attributed to the sacred prophets and seers of old. As such music is not produced in these latter days, so such utterances are heard no more. As in the one case men beloved of God may have been permitted to catch a glimpse of heaven, or to hear the words of eternal wisdom and give them to man, it is not difficult to believe that the ancient music of the church may be glimpses of the sublime chords that are heard in heaven. Every one knows how charming is music heard from a distance, and coming from you know not where. You catch three or four bars borne on the air, then there is silence, and the silence makes the music, when it does come, all the more delicious. With what ecstacy must one hear in his own soul the echo of a strain sent to him from God. All other gifts, all other intuitions by the side of this, sink into utter insignificance and worthlessness. It is a combination of the highest poetry, the most exalted religion, and the most devoted worship.

It is hard to believe, inasmuch as the letters of a written lauguage have been known for so many centuries, that the method of writing music, as now used, has been employed for only a little more than eight hundred years. St. Ambrose used the Greek method heretofore described, but Guido of Arrezzo, a small town in Tuscany, a Benedictine monk, in the early part of the tenth century, having given himself up to the study of music, became dissatisfied with such notation and devised the present style, or, at least, was the first to use the lines and spaces with little points to indicate the notes. He also added to the ancient system a bass note answering to the G or *sol* in our *fa* or bass clef. He called this note by the Greek name of the letter G, or *gamma*, and hence this series of sounds in the scale is called the *gamut*.

Up to the time of Guido something had been known of harmony, but it was very little. It is certain that among the ancients, what they called harmony, was limited to that agreeable succession of sounds which is now called air or melody. The Chinese and other far eastern nations know nothing to this day of this invention, as any one may ascertain, if he is ever present at an entertainment given by those of the race named or their neighbors, the Japanese. To appreciate it thoroughly or understand all of its beauties requires a highly cultivated ear, or an organization naturally attuned to it. Guido wrote upon this subject and was the first to fix the attention of musicians to it. He was largely assisted in his efforts in this direction, by the organ which had been introduced into France two hundred years or thereabouts before his time. Being played with keys, the production of simultaneous sounds became easy and the beautiful effect of their concordant union became known. But all of the capabilities of the instrument were not fully understood and appreciated until Guido pointed them out.

The organ, the most complete and magnificent of musical instruments, or the principle of its construction, has been known as long as civilized man has been known, although the method of its construction and use was very different from what it has latterly become.

Franco of Cologne, who lived about the time of Guido, or soon after, also wrote extensively on harmony and measured music, and these two may very properly be considered the fathers of modern music and the authors of the musical notation now in use, all subsequent changes being merely modifications of their inventions, rendered necessary in the improvement of the art.

It was a long time after these masters, that music passed out of the control of the church into secular use. The coming of such an event was indicated in 1540, so modern as to be after the discovery of America, half a century. In the year named, the first oratorio was publicly presented, called so from the fact of its being performed by an organization at Rome known as the Congregation of the Priests of the Oratory. These oratorios consisted of sacred interludes written by conspicuous poets of the time, and successfully turned to pious account, the theatrical enthusiasm then prevailing. It was but a short step from these to the opera in which the themes were not exactly of a sacred character, the first work of this kind being presented about 1597, its title being "Daphne."

Since that time there has been a constant improvement and advance in the art of music, its annals being glorified by the works of such masters as Mozart, Handel, Haydn, Beethoven and Mendelssohn, and the popular apprehension has been reaching beyond and above its simpler and commoner forms into its higher, more complex and more exalted flights.

There is a kind of composition, emanation or creation which is to true music something as are proverbs or slang expressions to literature. These are popular melodies, to which are sung words that catch the ear from some sentiment that is striking or prominent. They all have their influence and life, brief though they may be, and some are so peculiar and striking that they seem readily to become national in the country where they first appear, and are recognized as such the world over. But it would be impossible to tell where some of these first originated. They may have been floating around the world, here a strain and there a strain, for centuries, as it is said, some of the simple succession of sounds that a mother sings as a lullaby to her child now, were first sung in those countries in Asia where the Aryan race had its cradle and origin ages and ages ago. Travelers from civilized countries, in remote regions, have heard songs that were indigenous there but which resembled, in a marked manner, those they had heard in other and far distant quarters, between which two places the means of communication had only recently been opened. Besides helping to show the common origin of the race of man, these things also go to make manifest the fact, that music in its simplest form, in melody or tune, was among the earliest of man's recreations or methods of expression.

As we could not very well, without weakening the power of our language, do away with all proverbs and as what may be "slang" to-day, may become classical to-morrow, so these "popular melodies" as they are called, fill an important place. They assist the musical life of a people, give expression to the prevailing sentiment of the time and some of them really deserving to be perpetuated, have an enlivening influence on the religious or political life of a nation that is felt for years and years.

Instances of this are afforded by some of the "Moody and Sankey" hymns, and by patriotic airs. Who has not seen a crowded audience almost rise to its feet, when a few opening notes indicate to them that the "Sweet By-and-By" is to be sung or played; or where is the Frenchman who will not glow with enthusiasm over the notes of the "Marseillaise Hymn" be it never so roughly or hoarsely sung? Let an American far away from home and absent for any length of time, hear the piping notes of "Yankee Doodle," and if he can keep the tears from his eyes at the memories the melody excites, hard-hearted and unfeeling is he indeed.

Does it not suggest itself that if such effects can be produced by simple things, to what an ecstacy of delight one could be exalted by the perfect performance of the work of a master, who holds all the powers, resources and capabilities of the art of music under his complete control? The one, it seems to me, is but pulling at one string of your heart, while the other plays upon all of them at once.

